# Cysteine Mutations in the Ebolavirus Matrix Protein VP40 Promote Phosphatidylserine Binding by Increasing the Flexibility of a Lipid-Binding Loop

**DOI:** 10.3390/v13071375

**Published:** 2021-07-15

**Authors:** Kristen A. Johnson, Nisha Bhattarai, Melissa R. Budicini, Carolyn M. LaBonia, Sarah Catherine B. Baker, Bernard S. Gerstman, Prem P. Chapagain, Robert V. Stahelin

**Affiliations:** 1 Department of Chemistry and Biochemistry, University of Notre Dame, Notre Dame, IN 46556, USA; kjohns33@alumni.nd.edu (K.A.J.); Melissa.Budicini@UTSouthwestern.edu (M.R.B.); carolynlabonia@gmail.com (C.M.L.); sbaker6@alumni.nd.edu (S.C.B.B.); 2 Department of Physics, Florida International University, Miami, FL 33199, USA; nbhat006@fiu.edu (N.B.); gerstman@fiu.edu (B.S.G.); chapagap@fiu.edu (P.P.C.); 3 The Biomolecular Sciences Institute, Florida International University, Miami, FL 33199, USA; 4 Department of Medicinal Chemistry and Molecular Pharmacology and the Purdue Institute of Inflammation, Immunology and Infectious Disease, Purdue University, West Lafayette, IN 47907, USA

**Keywords:** ebolavirus, lipid-protein interaction, matrix protein, membrane bilayer, membrane binding, oligomerization, phosphatidylserine, plasma membrane, viral budding, VP40

## Abstract

Ebolavirus (EBOV) is a negative-sense RNA virus that causes severe hemorrhagic fever in humans. The matrix protein VP40 facilitates viral budding by binding to lipids in the host cell plasma membrane and driving the formation of filamentous, pleomorphic virus particles. The C-terminal domain of VP40 contains two highly-conserved cysteine residues at positions 311 and 314, but their role in the viral life cycle is unknown. We therefore investigated the properties of VP40 mutants in which the conserved cysteine residues were replaced with alanine. The C311A mutation significantly increased the affinity of VP40 for membranes containing phosphatidylserine (PS), resulting in the assembly of longer virus-like particles (VLPs) compared to wild-type VP40. The C314A mutation also increased the affinity of VP40 for membranes containing PS, albeit to a lesser degree than C311A. The double mutant behaved in a similar manner to the individual mutants. Computer modeling revealed that both cysteine residues restrain a loop segment containing lysine residues that interact with the plasma membrane, but Cys^311^ has the dominant role. Accordingly, the C311A mutation increases the flexibility of this membrane-binding loop, changes the profile of hydrogen bonding within VP40 and therefore binds to PS with greater affinity. This is the first evidence that mutations in VP40 can increase its affinity for biological membranes and modify the length of Ebola VLPs. The Cys^311^ and Cys^314^ residues therefore play an important role in dynamic interactions at the plasma membrane by modulating the ability of VP40 to bind PS.

## 1. Introduction

Ebolavirus (EBOV) is a group of six negative-sense RNA viruses, four of which are known to cause severe hemorrhagic fever in humans with a high fatality rate. The most serious EBOV outbreak thus far (2013–2016 in Western Africa) resulted in more than 28,000 cases and over 11,000 fatalities. Even more recently, an EBOV outbreak in the Democratic Republic of Congo lasted from 2018–2020 with over 3400 documented cases and greater than 2200 deaths. A smaller outbreak also occurred in the Democratic Republic of Congo in 2021, resulting in 12 probable cases and 6 deaths. Research focusing on the development of vaccines and antibody therapies against the EBOV glycoprotein has shown great promise with recent FDA approval of an EBOV vaccine and monoclonal antibody cocktail [1,2]. Despite these successes there is still a lack of small molecule therapeutics for infected patients and the efficacy of the vaccine and antibodies against the different ebolavirus strains is not well known. Further, antibody escape mutants have been detected when EBOV was passed through animals [3] and a watch list of potential glycoprotein mutations that may evade antibody control has been compiled [4]. Thus, investigation of the mechanisms by which ebolaviruses replicate in human cells and identification of alternative drug targeting strategies is warranted [5,6].

The EBOV genome features seven genes encoding only eight proteins, but the transformative properties of the matrix protein VP40 allows it to perform multiple functions in the virus life cycle [7,8,9]. VP40 is necessary for the transcriptional regulation of viral gene expression during infection [7], the formation of enveloped virus particles and the budding of these particles from the host cell plasma membrane [8,9,10,11,12]. The multifunctional properties of VP40 depend on its flexibility, which is conferred by specific flexible regions within the polypeptide backbone (7). These regions facilitate conformational changes that allow VP40 to bind RNA [7,13] and lipids [14,15,16], assemble into various oligomers and interact with multiple host proteins to achieve transport to the plasma membrane and thus enable viral budding [8,9,10,11,12,14,15,16,17,18,19,20,21,22,23,24,25,26]. VP40 is sufficient to form virus like particles (VLPs) that bud from the plasma membrane of cells independent of the other EBOV components [8,9,10,11].

VP40 binds to anionic lipids at the plasma membrane to form the host-derived lipid envelope of the virus particle [8,14,15,16,27,28,29]. VP40–lipid binding is also essential for the formation of VP40 oligomers [8,14,15,26,27,28,30,31] and the matrix sheet of large oligomers [8,14,15,27] that form at the plasma membrane in both infected and cells expressing VP40 [32]. VP40 interacts with the plasma membrane via its C-terminal domain (CTD) [7,8,15,16,27,28,31] and forms oligomers via interactions involving its CTD [7,32]. VP40 oligomers are formed when the protein binds to the anionic lipid phosphatidylserine (PS) in vitro or in live cells [8,14,16,26,27,28,30]. Lipid-induced VP40 oligomerization allows the dissociation of the NTD and CTD, which normally stabilize the protein in the closed dimer conformation [7,31]. The binding of VP40 to the plasma membrane is mediated by the presence of PS and the low abundance of phosphatidylinositol 4,5-bisphosphate (PI(4,5)P_2_). Both lipids are needed to form VLPs, but they have separate roles: PS promotes initial plasma membrane recruitment and oligomer formation [8,14,16] whereas PI(4,5)P_2_ is needed for extensive VP40 oligomerization and oligomer stability [15,33]. When lipid binding is abolished in cells, VP40-dependent budding is significantly reduced [8,14,16,27,28].

VP40 has two highly-conserved cysteine residues in the CTD. In many EBOV strains, they form a C-(X)_2_-C motif which is typically associated with metal-binding proteins and oxidoreductases [34]. Cysteine residues play an important role in protein structure by forming disulfide bonds, and they also interact with nucleic acids and histone proteins via zinc finger motifs [35]. However, the crystal structure of the Zaire ebolavirus VP40 (4LDB) revealed that the cysteine residues are not engaged in a disulfide bond [7]. This is unsurprising, because VP40 is a cytosolic protein and most such proteins do not form disulfide bonds because the reduced form is maintained by thioredoxin proteins [34]. Cysteine residues are comparatively rare in higher eukaryotic proteins and tend to fulfil important functions, particularly in the context of a C-(X)_2_-C motif. We, therefore, hypothesized that the two cysteine residues in VP40 must be important in the viral infection cycle, but the role of these residues has not been investigated in detail.

To determine how these highly-conserved cysteine residues influence the function of VP40, we tested the lipid-binding and oligomerization properties of VP40 single and double mutants in which the cysteine residues were replaced with alanine. We investigated the behavior of the mutants in vitro and in cells, measured VLP formation and filament length and performed molecular dynamics simulations to investigate the role of these residues at the molecular level.

## 2. Materials and Methods

### 2.1. Molecular Biology

Site-directed mutagenesis was carried out using the QuikChange II XL Site-Directed Mutagenesis Kit according to the manufacturer’s instructions (Agilent Technologies, Santa Clara, CA, USA). Primers were synthesized by Integrated DNA Technologies (Coralville, IA, USA) according the specifications in the QuickChange kit. The C311A mutation was introduced using forward primer 5′-CACACAGGATGCTGACACGTGTCATTCT CCTGC-3′ and reverse primer 5′-GCAGGAGAATGACACGTGTCAGCATCCTGTGTG-3′. The C314A mutation was introduced using forward primer 5′-CACACAGGATTGT GACACGGCTCATTCTCCTGC-3′ and reverse primer 5′-GCAGGAGAATGAGCCGT GTCACAATCCTGTGTG-3′. The C311A/C314A double mutant was introduced using forward primer 5′-CACACAGGATGCTGACACGGCTCATTCTCCTGC-3′ and reverse primer 5′-GCAGGAGAATGAGCCGTGTCAGCATCCTGTGTG-3′ with the single mutant as template DNA. Mutations were verified by Sanger sequencing at the Notre Dame sequencing facility and the sequence data were analyzed using 4Peaks (Nucleobytes, Aalsmeer, The Netherlands).

### 2.2. Protein Expression and Purification

The His_6_-VP40-pET46 construct was introduced into *Escherichia coli* Rosetta BL21 DE3 cells according to the manufacturer’s instructions (Novagen/Merck, Darmstadt, Germany). Transformed bacteria were grown at 37 °C until an OD_600_ of 0.6–0.9 was achieved. Protein expression was induced with 1 mM IPTG at room temperature for 4–6 h. Bacteria were pelleted at 4000× *g* and stored at −20 °C before protein extraction as previously described in detail [15]. The eluted protein was purified by size exclusion chromatography to isolate VP40 dimers from the dimer/octamer mixture. The protein concentration was determined using a Pierce BCA assay (Thermo Fisher Scientific, Waltham, MA, USA) and stored at 4 °C in 10 mM Tris (pH 8.0) containing 300 mM NaCl for up to 2 weeks.

### 2.3. Liposome Pelleting Assay

Liposomes were prepared with the following compositions: (1) control liposomes, a 49:49:2 ratio of 1,2-dipalmitoyl-*sn*-glycero-3-phosphocholine (DPPC), cholesterol and 1,2-dioleoyl-*sn*-glycero-3-phosphoethanolamine-N-(5-dimethylamino-1-naphthalenesulfonyl (dansylPE); (2) PI(4,5)P_2_-containing liposomes, a 46.5:46.5:5:2 ratio of DPPC, cholesterol, PI(4,5)P_2_ and dansylPE, and (3) PS-containing liposomes, a 29:29:40:2 ratio of DPPC, cholesterol, 1-palmitoyl-2-oleoyl-*sn*-glycero-3-phospho-L-serine (POPS) and dansylPE. All lipids were purchased from Avanti Polar Lipids, Inc. (Alabaster, AL, USA). The lipids were dried under a nitrogen gas stream and stored at −20 °C. Lipid films were hydrated with 250 mM raffinose pentahydrate in 10 mM Tris (pH 7.4) containing 150 mM NaCl and liposomes were extruded through a 200 nm filter. Dynamic light scattering was used to confirm their size. Raffinose liposomes were then diluted and added to the protein/buffer mixture for the binding experiment. Protein and liposomes were incubated for 30 min at room temperature then centrifuged to pellet the liposomes (75,000× *g* for 30 min at 22 °C). Supernatants were removed and the pellets were re-suspended under a UV wand to visualize the 2% dansylPE included in the liposomes. Supernatant and pellet fractions were loaded onto polyacrylamide gels and fractionated by SDS-PAGE, and protein band density in each lane was determined using ImageJ software. All liposomes were used on the day of preparation and the raffinose buffer was made fresh each day.

### 2.4. Molecular Dynamics Simulations

The crystal structure of the VP40 dimer was acquired from the Protein Data Bank (PDB ID: 4LDB). The protein and plasma membrane complex was set up using the Charmm-Gui web server [36,37]. The plasma membrane contained 1-palmitoyl-2-oleoyl-*sn*-phosphatidylcholine (POPC), 1-palmitoyl-2-oleoyl-*sn*-phosphatidylethanolamine (POPE), POPS, palmitoylsphingomyelin (PSM), palmitoyl-oleoyl-phosphatidyl-inositol (POPI) and cholesterol. The lipid composition in the lower leaflet was set to 11:32:17:9:10:21 POPC:POPE:POPS:POPI:PSM:cholesterol. There were 149 lipids on the upper leaflet and 151 on the lower leaflet. The system was solvated using TIP3 water molecules and neutralized with counter ions. The total system consisted of ~122,000 atoms. All-atom molecular dynamics simulations were performed using NAMD v2.12 [38] with the CHARMM36 force field [39]. The particle mesh Ewald (PME) method [40] was used to calculate long-range electrostatic interactions and the SHAKE algorithm was used to constrain the covalent bonds. The system was minimized for 10,000 steps with six-step equilibration in Charmm-gui [37]. A Nosé–Hoover Langevin piston was used to control the pressure with a piston period of 50 fs decay and 25 fs. The Langevin temperature coupling with friction coefficient of 1 ps^−1^ was used to control the temperature. All production runs included a 2-fs time step. Models were visualized and rendered with Visual Molecular Dynamics (VMD) [41].

### 2.5. Cell Culture and Transfection

COS-7 cells were maintained and transfected as previously described [15].

### 2.6. Confocal Imaging

A Zeiss 710 laser scanning confocal microscope was used to visualize enhanced green fluorescent protein VP40 (EGFP-VP40) phenotypes in live cells. Number and brightness data were acquired on an Olympus FV2000 microscope and the data were analyzed using SimFCS [42,43,44].

### 2.7. Fluorescence Recovery after Photobleaching (FRAP)

FRAP analysis was carried out as previously described with some modifications [45]. A 2.2-μm^2^ region of interest was used to bleach EGFP with 50 iterations of 100% 488 nm laser power after three pre-bleach scans. Fluorescence recovery was measured for 30 s following the beaching. Data represent 20 images per construct collected over two different days and four independent experiments.

### 2.8. Scanning Electron Microscopy (SEM) Analysis

COS-7 cells were transfected for 12–16 h then scraped from the plates, pelleted by centrifugation (900× *g*, 6 min, room temperature), washed with PBS and suspended in primary fixative. The samples were prepared for SEM as previously described [15].

### 2.9. VLP Collection

VLPs were collected 24 h post-transfection as previously described [15]. Briefly, the supernatant of the transfected cells was collected and applied to a 20% sucrose cushion. Samples were centrifuged (100,000× *g*, 2 h, room temperature) and the VLP pellets were re-suspended in 150 mM ammonium bicarbonate. Cells were trypsinized from the plate and lysed with RIPA buffer (with protease inhibitors) for 1 h on ice with intermediate vortexing. Samples were centrifuged (25,000× *g*, 17 min, room temperature) and the soluble fraction was removed. The protein concentration in the cell lysate was determined using a Pierce BCA assay.

### 2.10. Western Blot Analysis

VLP samples and cell lysates were separated by SDS-PAGE (15 μg of protein per lane for the cell lysates, and five times the cell lysate volume for the VLP samples) and transferred to a membrane using a transblot device (BioRad, Hercules, CA, USA). VP40 was detected using primary antibody F56-6A1.2.3 (Thermo Fisher Scientific) and secondary antibody AB 6808 conjugated to horseradish peroxidase (Abcam, Cambridge, UK). GAPDH was detected using primary antibody AB 8245 (Abcam) and secondary antibody AB 6808 as above. Signals were detected using the enhanced chemiluminescence substrate according to the manufacturer’s instructions (Thermo Fisher Scientific). The percentage of budding was determined by FIJI analysis [46].

## 3. Results

### 3.1. VP40 Features Two Closely-Spaced Cysteine Residues in the CTD

**Figure 1:**
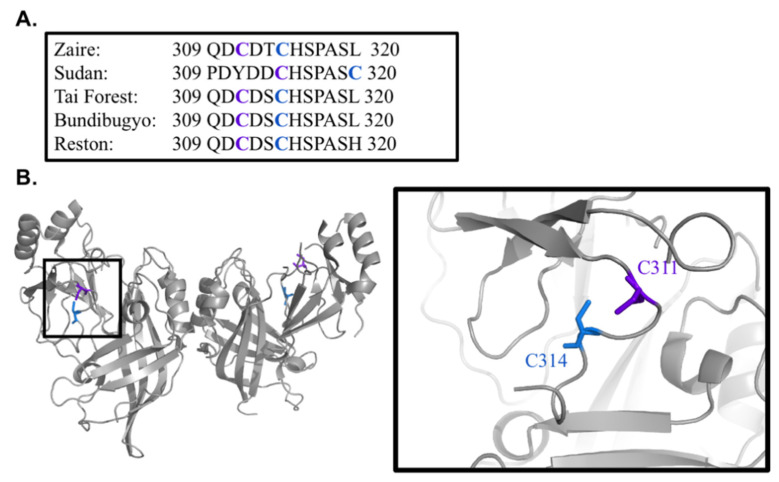
Ebolavirus VP40 cysteine residues are conserved. (**A**) With the exception of Sudan Ebolavirus, cysteine at 311 and 314 are conserved in ebolaviruses. (**B**) Structure of Ebolavirus Zaire VP40 (PDB ID: 4LDB) with C^311^ and C^314^ highlighted in the CTD loop region.

The VP40 polypeptide of the Zaire, Tai Forrest, Bundibugyo, and Reston ebolaviruses features conserved cysteine residues at positions 311 and 314, but these are displaced to positions 314 and 320 in the Sudan ebolavirus (Figure 1A). The crystal structure of Zaire ebolavirus VP40 (4LDB) reveals that these residues are located within a solvent-exposed, flexible loop on the side of the CTD (Figure 1B). The corresponding residues are not resolved in the Sudan ebolavirus VP40 structure (PDB ID: 4LD8), probably because they are also located within a flexible loop. To investigate the role of these cysteine residues, we replaced either or both residues with alanine and prepared two constructs for each mutant: a His_6_-VP40-pET46 construct for bacterial expression and protein purification, and an EGFP-VP40 fusion construct for expression in cell culture to facilitate imaging and budding experiments.

### 3.2. The Replacement of Cysteine with Alanine Increases the Affinity of VP40 for PS

**Figure 2:**
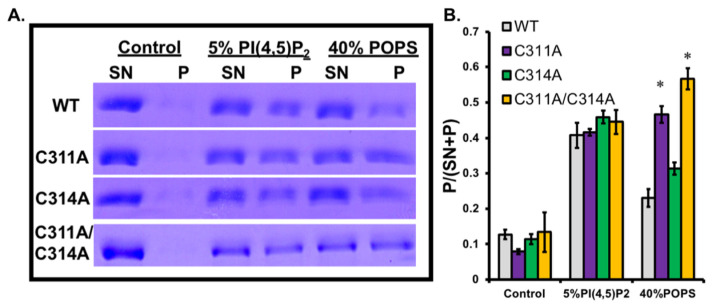
VP40 cysteine mutants have increased binding to the anionic lipid phosphatidylserine. (**A**) Representative SDS-PAGE of supernatant (SN) and pellet (P) samples from an large unilamellar vesicle (LUV) pelleting assay (control: DPPC:cholesterol:danyslPE (49:49:2), 5% PI(4,5)P_2_: (DPPC:cholesterol:PI(4,5)P_2_:dansylPE (46.5:46.5:5:2), or 40% POPS: DPPC:cholesterol:POPS:dansylPE (29:29:40:2)) with VP40-WT, VP40-C311A, VP40-C314A, and VP40-C311A/C314A. (**B**) Fraction of VP40 bound to lipids (P/(SN + P)) is shown as the average fraction bound over three independent experiments. Error bars represent the standard error of the mean, and * marks significance of a *p* value of 0.05 or less.

VP40 binds to PS and PI(4,5)P_2_ in the host cell plasma membrane [14,15]. To determine whether the cysteine residues are required for lipid binding (Figure 2), we expressed wild-type VP40 and the C311A and C314A mutants, and purified them for liposome pelleting assays. The His_6_-VP40-pET46 construct produces VP40 dimers and octamers in solution, so we separated them by size exclusion chromatography and used only the dimers for our experiments. Dimers are the main building block of VP40-plasma membrane lipid interactions in EBOV assembly [7,8,16,32].

The liposome pelleting assay allows the quantitative comparison of protein fractions that bind control liposomes and liposomes containing 40% PS or 5% PI(4,5)P_2_. SDS-PAGE analysis indicated the distribution of unbound protein in the supernatant and the fraction bound to the liposomes in the pellet (Figure 2A). The bound fraction for each mutant and each type of liposome is shown in Figure 2B. The C311A mutation resulted in a significant increase in affinity for PS, whereas the C314A mutation only caused a slight increase compared to wild-type VP40. The C311A/C314A double mutant showed the greatest increase in affinity for PS, approximately equivalent to the additive effect of both individual mutations. In contrast, there was no significant change in binding to PI(4,5)P_2_ in any of the mutants compared to wild-type VP40 (Figure 2B).

### 3.3. VP40 Cysteine Residues Confer Minor Effects on Protein Dynamics at the Plasma Membrane

**Figure 3:**
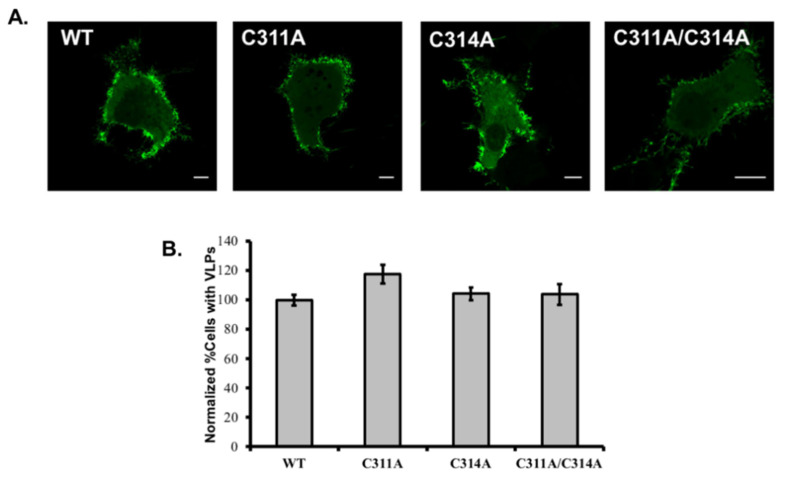
EGFP-VP40 and VP40 cysteine mutants cell phenotype. (**A**) Representative images of EGFP-VP40-WT, EGFP-VP40-C311A, EGFP-VP40-C314A and EGFP-VP40-C311A/C314A 12–14 h post transfection in COS-7 cells. Scale bars are 10 μm. (**B**) Cell populations expressing pre-VLPs, data was normalized to 100% for cells expressing EGFP-VP40-WT with detectable VLPs to more easily differ an increase (or not) in VLPs by each mutation. Bars represent the average value over three independent experiments and error bars represent ± standard error of the mean.

The VP40 C311A and C311/C314A mutants bound more strongly to PS, an anionic lipid enriched in the inner leaflet of the host cell plasma membrane, which is necessary for VP40 budding [14]. We therefore expressed EGFP-tagged VP40 constructs in COS-7 cells to observe the phenotype of the mutants. The population of cells producing VLPs 14 h post-transfection was 70% (SD = 7.9%, n = 3 independent experiments). Since C311A and the double mutant displayed an increase in PS binding compared to WT, we normalized the quantitative representation of WT cells with VLPs to more notably differ increases in VLP formation for mutants compared to WT. We counted the cells expressing/not expressing VLPs assembled from wild-type VP40 or the C311A, C314A and C311A/C314A mutants, and representative images are shown in Figure 3A. Mutant C311A caused a slight increase in the number of cells shedding VLPs from the plasma membrane, but this increase was not statistically significant, and there was no change in the case of mutant C314A or the double mutant (Figure 3B).

**Figure 4:**
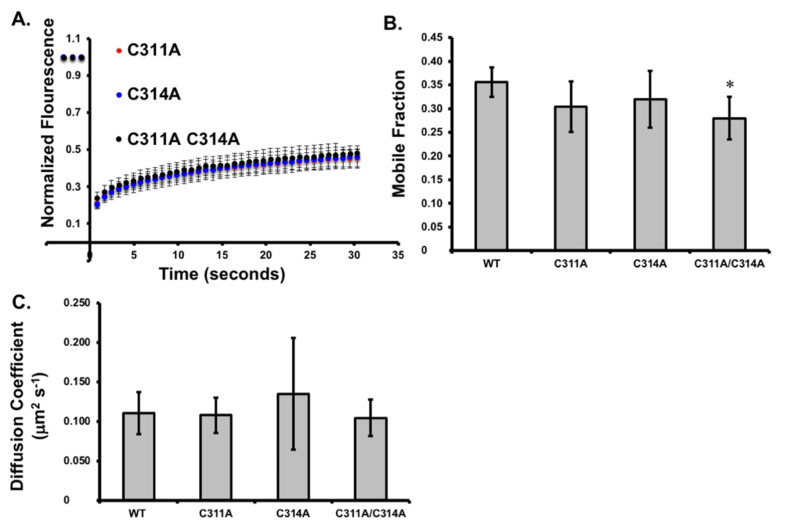
VP40 dynamics at the plasma membrane. (**A**) Fluorescence recovery after photobleaching (FRAP) plot of EGFP-VP40-WT and mutants in COS-7 cells. (**B**) The mobile fraction of EGFP-VP40-WT and mutants in COS-7 cells. (**C**) The diffusion coefficient of EGFP-VP40-WT and mutants was determined and plotted. Values represent the average value over at least three independent experiments and error bars represent ± standard error of the mean. Significance is marked with a *, determined as a *p* value of 0.05 or less.

We then investigated VP40 membrane dynamics by fluorescence recovery after photobleaching. The mean normalized FRAP profile for each construct is shown in Figure 4A and the mobile fraction is shown in Figure 4B. The mutant forms of VP40 showed a slower recovery after photobleaching and the mobile fraction was in each case lower than the 0.36 recorded for wild-type VP40. The decrease in the mobile fraction was not statistically significant for the individual mutations (*p* = 0.054 for C311A, *p* = 0.175 for C314A) but the mobile fraction of the double mutant C311A/C314A was reduced to 0.28, which was a statistically significant difference (*p* = 0.029). The diffusion coefficient of wild-type VP40 (0.110 μm^2^/s) was similar to that of 0.108 μm^2^/s for C311A, 0.135 μm^2^/s for C314A and 0.104 μm^2^/s for C311A/C314A (Figure 4C). These results show that the wild-type and mutant forms of VP40 show similar degrees of diffusion at the plasma membrane.

**Figure 5:**
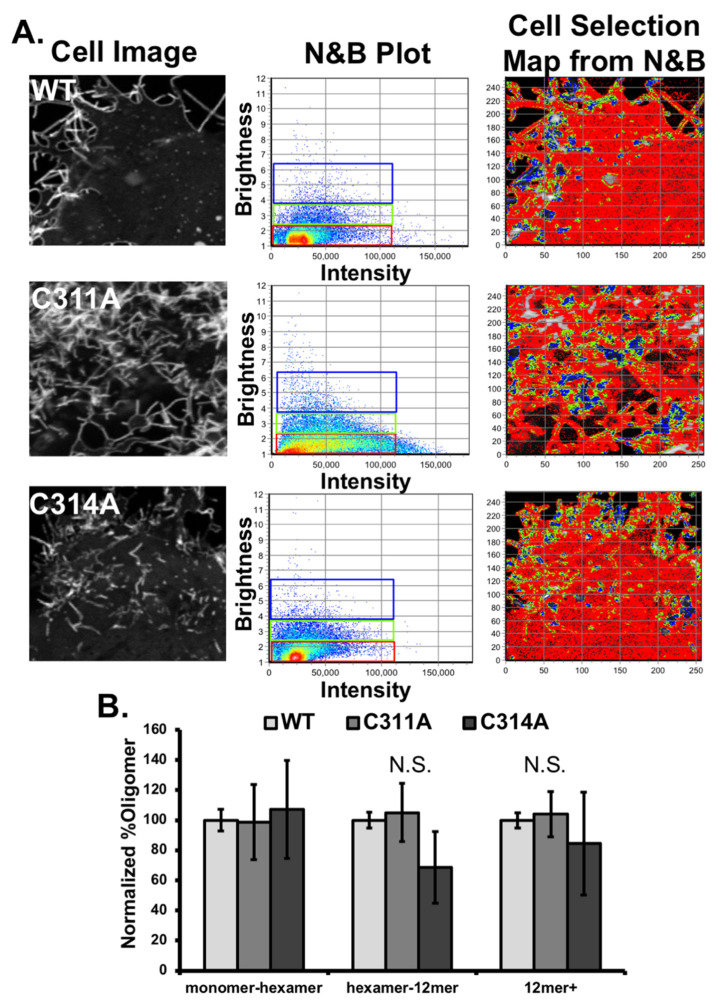
Assessment of VP40 and cysteine mutant oligomer formation in cells. (**A**) Representative images from the number and brightness experiment to determine oligomer formation in live cells. Left panel are raw images of WT and mutants from confocal microscopy. The middle panel are brightness (y-axis) versus intensity plots (x-axis) from raw images on the left as processed by the SIMFCS software. A value of 1.1 on the y-axis corresponds to the brightness value of a VP40 monomer and each additional VP40 unit corresponds to an addition of 0.1 on the y-axis. Three representative oligomer distributions were selected as representations shown with corresponding colors in the images on the panels on the right (red = monomer − 12 mer, green = 12 mer–36 mer, blue > 36 mer). (**B**) Oligomer formation in cells normalized to EGFP-VP40-WT. No significant changes were observed over three independent experiments. Bar graph is plotted (±the standard error of the mean) for three different (and common) VP40 oligomer distributions (monomer-hexamer, hexamer-12mer and 12mer and greater).

**Figure 6:**
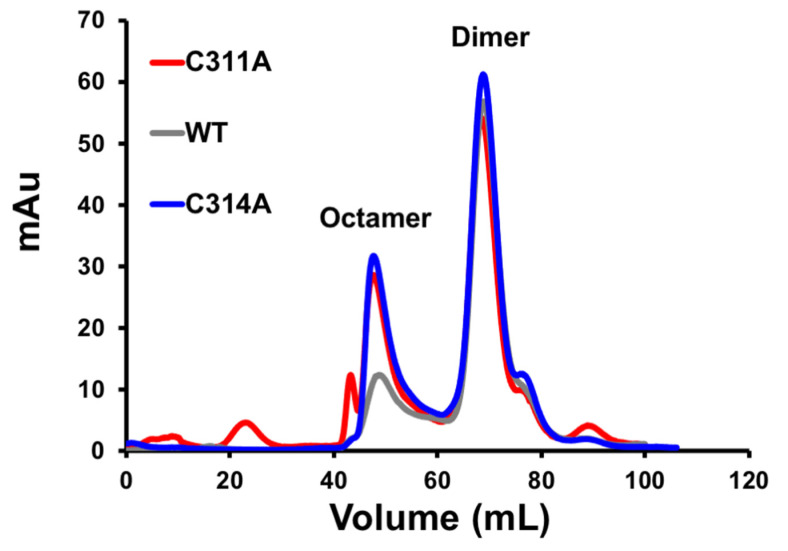
Dimer and octamer assessment of VP40 and single cysteine mutations in vitro. Size exclusion chromatogram trace of WT, C311A, and C314A purified VP40 protein shows similar formation of the dimer in solution compared to WT (only dimers used for lipid-binding assays). C311A and C314A both displayed an increased propensity towards the octamer form compared to WT VP40. The double mutant, C311A/C314A is not shown but demonstrated similar dimer and octamer peaks analogous to the single mutants.

Oligomerization of VP40 at the plasma membrane inner leaflet is an essential step in assembly and budding of EBOV VLPs. To determine if cysteine mutations of VP40 altered the oligomerization state of VP40, we assessed VP40 oligomerization in cells and in vitro (Figure 5 and Figure 6). Notably, there was no significant difference detected in VP40 oligomers in cells (Figure 5A,B) and the cysteine mutants had a similar fraction of dimer compared to the WT protein when purified from *E. coli* (Figure 6). While the dimer peaks for WT and the single cysteine mutations were similar in vitro, both cysteine mutations favored a higher amount of VP40 octamer detection (Figure 6). This suggest the cysteine mutations may slightly destabilize the N- and C-terminal domain interfaces in the VP40 dimer and favor an increase in octamer formation [7,13].

Number and Brightness analysis (N and B) was used to determine oligomerization of each VP40 construct at the surface of the cell [14,15,27,28,43]. N and B analysis allows for detection of approximate oligomerization state of a GFP-tagged protein when the microscope detection system is calibrated for the brightness of a GFP monomer. Previous imaging using this technique has allowed for the detection of mutant vs WT-VP40 oligomerization differences with respect to changes in plasma membrane binding [14,15,27,28,43]. There were no significant differences in VP40 oligomerization with WT, C311A, or C314A after three independent experiments. Representative images with corresponding N and B plots and cell plots are shown in Figure 5A. The population of monomer-hexamer, hexamer-12mer, and 12mer+ were normalized to the value of WT and plotted as shown in Figure 5B. Since the oligomerization state of C311A was similar to WT the double mutant was not analyzed in this live cell assay.

### 3.4. Molecular Dynamics Studies Show That Cysteine Residues Regulate the Position of a Lipid-Binding Loop

**Figure 7:**
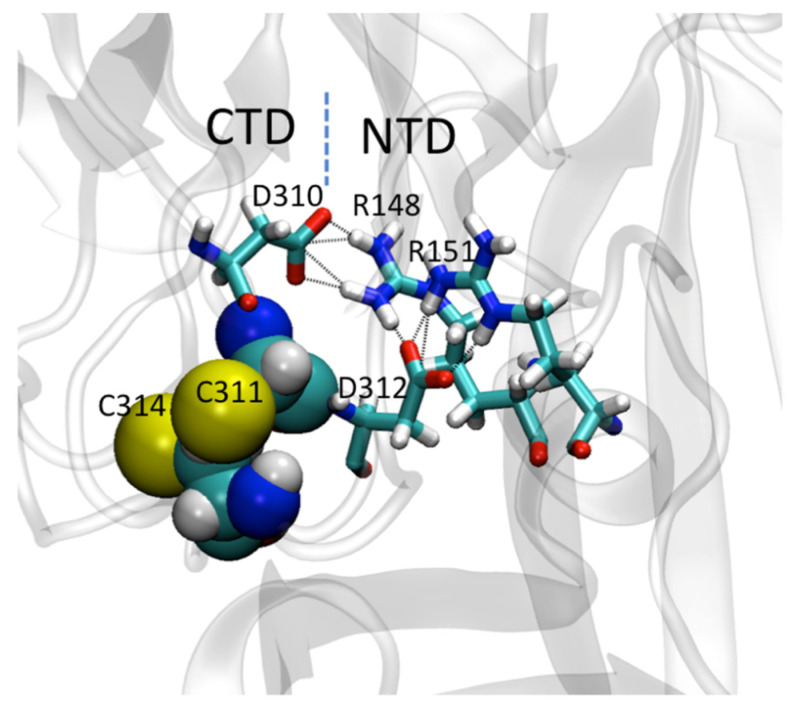
Molecular dynamics simulations reveal interdomain salt-bridges between Asp and Arg residues in the CTD and NTD, respectively. Aspartate residues (310 and 312) on either side of Cys^311^ are involved in the interdomain salt-bridges between N-and C-terminal domains.

**Figure 8:**
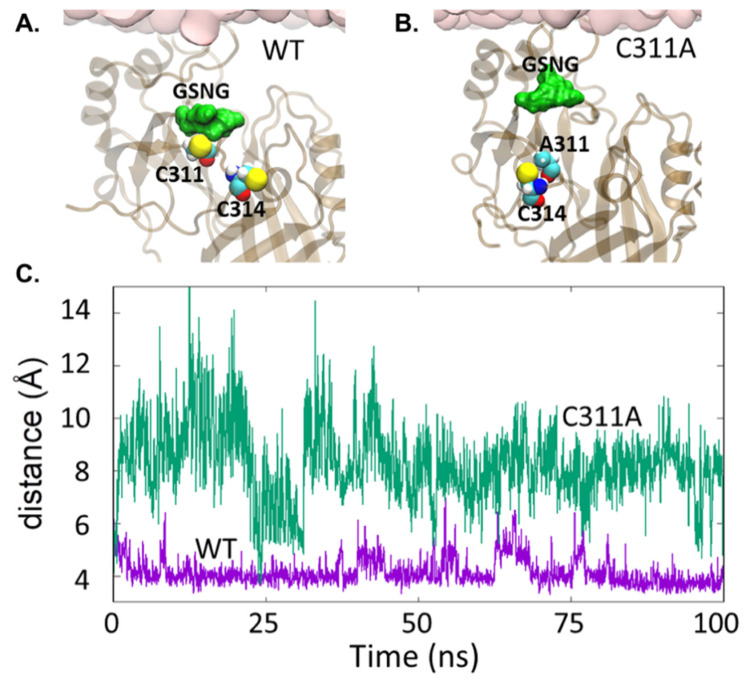
Molecular dynamics simulations demonstrate Cys^311^ restrains a CTD motif, whereas C311A enhances the CTD motif flexibility and C311A membrane binding. (**A**) Cys^311^ interacting with the two polar residues (Ser^199^, Asn^200^) in the GSNG motif. (**B**) C311A mutation enhances the flexibility of the GSNG motif, which underlies the CTD lipid binding surface of VP40. (**C**) Distance between the C_α_ atoms of residues 201 and 311 (purple: WT, green: C311A).

Molecular dynamics simulations showed that both VP40 cysteine residues play an important role in the flexibility of the CTD, but Cys^311^ is particularly important because it interacts with Ser^199^ and Asn^200^ in the 198-GSNG-201 loop. The latter resembles the motif GxxG, which is functionally important RNA binding and protein–ligand interactions in other protein systems [47,48,49]. The interaction between Cys^311^ and Ser^199^/Asn^200^ appears to facilitate the formation of a salt bridge between the NTD and CTD by promoting interactions between flanking aspartate residues (Asp^310^, Asp^312^) in the CTD and two arginine residues (Arg^148^, Arg^151^) in the NTD (Figure 7). Interactions between the NTD and CTD residues of VP40 are required for domain association, oligomerization, and plasma membrane localization [31]. The C311A mutation alters the distance between the C_α_ atoms of residues 311 and 201. The GSNG loop region extends toward the membrane during the simulation time (Figure 8), which helps to explain the greater affinity of the cationic CTD residues for PS in the C311A mutant, given that lysine residues near the GSNG motif may facilitate interactions with PS at the plasma membrane [7,16] and are necessary for interactions with PS-containing lipid vesicles [16].

### 3.5. The C311A Mutation Increases VLP Filament Length

**Figure 9:**
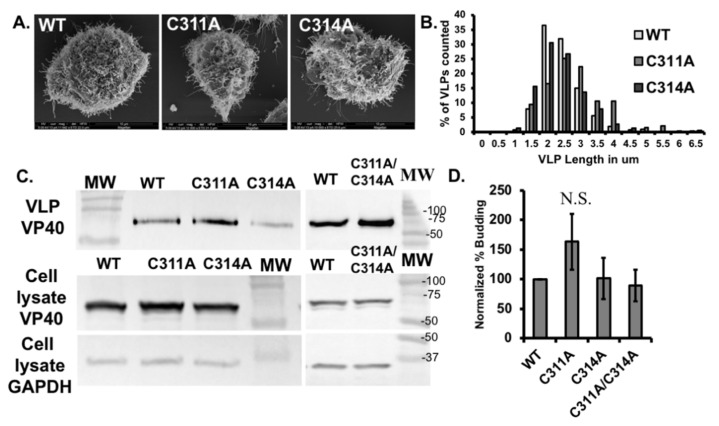
VLP filament length and budding efficiency assessment for WT-VP40 and cysteine mutations. (**A**) Representative scanning electron micrographs of EGFP-VP40-WT, and single mutants to show VLPs on the surface of the cell. (**B**) Histogram of VLP length for EGFP-VP40-WT and single mutants, values are represented as the percentage of the population of VLPs at each length bin for each construct. (**C**) Western blot of VP40 in the cell lysate and VLP samples. GAPDH was used as the loading control for the cell lysates. (**D**) Normalized budding efficiency for EGFP-VP40-WT and single Cys mutants. Average values are normalized to WT budding efficiency and represent at least three independent experiments. Error bars are ± standard error of the mean. N.S. = not significant.

When VP40 is expressed in mammalian cells, VLPs form at the surface of the plasma membrane. This process can be observed by confocal microcopy (in cells transfected with fluorescent VP40 constructs) or scanning electron microscopy (SEM) to visualize structures on the cell surface directly [15,31]. Here we used SEM to visualize the VLPs on the surface of COS-7 cells transfected with wild-type VP40 and the single cysteine mutants (Figure 9A). We measured the length of the VLPs using FIJI and plotted the values to determine the distribution of particle lengths. This revealed that the C311A VLPs were longer than the wild-type and C314A particles, reflecting the greater abundance of particles ≥ 3 µm in length and the relative depletion of particles 2 µm in length (Figure 9B).

To determine whether the enhanced PS binding observed in the mutants affected VP40 budding efficiency, we transfected cells with wild-type VP40 and the mutants and collected the VLPs and cell lysates for testing 24 h post-transfection. We measured the levels of VP40 in each fraction by western blot, with glyceraldehyde 3-phosphate dehydrogenase (GAPDH) as the loading control (Figure 9C). The C311A mutant (n = 4) showed a slight increase in budding efficiency compared to WT VP40 (n = 7), although the difference was not statistically significant. The budding efficiencies of C314A (n = 4) and double mutant (n = 3) were similar to that of WT VP40 (Figure 9D) indicating that despite increases in PS binding by C311A and C311A/C314A, this did not significantly contribute to changes in VLP formation.

## 4. Discussion

The lipid-binding properties of VP40 are required for the efficient budding of EBOV from the plasma membrane of mammalian cells [16,33,50]. In this study, we found that the two conserved cysteine residues in VP40 influence its interactions with membranes containing PS. The C311A mutation alone or in combination with C314A significantly enhanced the interaction between VP40 and PS, whereas the C314A mutation alone had a limited effect and there was no significant difference compared to wild-type VP40. Molecular dynamics simulations provided a mechanistic explanation, showing that Cys^311^ plays a predominant role by interacting with the 198-GSNG-201 motif, which is adjacent to the Lys^224^ and Lys^225^ residues of the membrane-binding loop responsible for key contacts with the plasma membrane [7,16,33]. These residues are absolutely required to bind PS in vitro [16]. The polar residues Ser^199^ and Asn^200^ in the 198-GSNG-201 motif interact with Cys^311^, restricting the ability of the loop region to extend towards the membrane surface (Figure 8A). However, this restriction is lifted in the C311A mutant (Figure 8B) as reflected by the greater distance between the C_α_ atoms of residue 311 and Gly^201^ in the GSNG loop (Figure 8C). Interestingly, this loop opens and closes intermittently in the absence of membrane interactions (Supporting Information, Supplementary Video S1).

PS becomes exposed on the external surface of VP40 VLPs following the accumulation of VP40 at the inner leaflet of the plasma membrane [15]. The PS content of the plasma membrane determines the efficiency of EBOV assembly and budding, and reducing the PS content by 35–40% significantly inhibits VLP formation and reduces the number of detectable budding sites [15]. However, it is unclear whether increasing the PS content of the plasma membrane or the affinity of VP40 for PS would increase the number of VLPs produced. We observed a higher efficiency of budding in mutant C311A, whereas mutant C314A and the double mutant were similar to wild-type VP40, suggesting that C314A has a minimal effect alone and may counter the effect of C311A in the double mutant.

The VLPs derived from mutant C311A were consistently and significantly longer than wild-type particles, which may reflect the enhanced PS binding and/or changes in the interdomain or interdomain contacts of the VP40 monomers. VP40 mutations were detected throughout the 2013–2016 EBOV outbreak, and are also routinely detected when EBOV was passed through animals [51,52]. The functional consequences of these mutations are largely unknown, but a mutation affecting Cys^311^, its interacting residues, or residues adjacent to this region, could increase the affinity of VP40 for PS in the plasma membrane during infections with the live virus.

Some cysteine residues or motifs containing multiple cysteines are known to bind nucleotides [35]. VP40 forms an octameric ring that binds RNA, but residues in the NTD make contact with the nucleotides so the cysteine residues in the CTD are unlikely to be directly involved [7,53]. However, we considered the possibility that the cysteine residues might influence this process indirectly, by promoting the formation of oligomers. We found that the ability of VP40 mutants to form dimers in vitro was unaltered, but the octamer fractions of all mutations were formed in higher proportions compared to WT VP40 (Figure 5). Number and brightness analysis indicated that there was no significant difference in oligomerization between wild-type VP40 and the single and double mutants at the plasma membrane. Additionally, EGFP-VP40 fusion proteins localized to the plasma membrane as anticipated, with no evidence for nuclear or perinuclear localization in cells transfected with mutant VP40 constructs. Our results were consistent across the in vitro and cellular experiments, but future research is needed to determine whether the cysteine residues are involved in the transcriptional regulation of viral genes (i.e., the role of the VP40 octamer) during the infection cycle.

In HIV, the mutation of a cysteine residue in the nucleocapsid protein P7 abolished the ability of the protein to bind nucleotides without affecting the efficiency of budding, but the resulting virions were non-infectious [54]. The role of VP40 in transcriptional regulation is not completely understood, but the wild-type protein reduces the rate of transcription by ~70% [7]. The R134A mutant, which lacks the ability to bind RNA, can only reduce the rate of transcription by ~30%, but nevertheless retains its budding ability [7]. If Cys^311^ and/or Cys^314^ are important for nucleotide binding or transcriptional regulation via a zinc finger motif, the C311A and C314A mutants might be considered consistent with the R134A phenotype. In previous structural studies of the VP40 octameric ring, CTD residues have not been implicated in nucleotide binding because the CTD is highly flexible and is not resolved in the available octameric ring structures. Therefore, further research is needed to investigate the potential metal and/or nucleotide binding abilities of the VP40 C-(X)_2_-C motif.

## 5. Conclusions

Taken together, our results show that cysteine-to-alanine mutations in the CTD of VP40 enhance the ability of the protein to bind membranes containing PS and increase the filament length of VLPs, without significantly affecting budding efficiency. Computer simulations suggest that the cysteine residues regulate the position of a membrane-binding loop, highlighting the importance of examining individual VP40 mutations for their effect on EBOV assembly, budding and transcriptional regulation.

## Data Availability

The majority of data are contained within the research article. Additional data are available upon request by contacting the corresponding author.

## Supplementary Materials

The following are available online at https://www.mdpi.com/article/10.3390/v13071375/s1, Video S1: Molecular dynamics simulations reveal the 198-GSNG-201 loop in VP40 is found to open and close intermittently in the absence of membrane.
